# *Lactiplantibacillus plantarum* L47 and inulin affect colon and liver inflammation in piglets challenged by enterotoxigenic *Escherichia coli* through regulating gut microbiota

**DOI:** 10.3389/fvets.2024.1496893

**Published:** 2024-11-27

**Authors:** Jingna Miao, Leihong Cui, Hui Zeng, Meixin Hou, Jingxuan Wang, Suqin Hang

**Affiliations:** Laboratory of Gastrointestinal Microbiology, National Center for International Research on Animal Gut Nutrition, Nanjing Agricultural University, Nanjing, China

**Keywords:** weaned piglet, *Lactiplantibacillus plantarum* L47, inulin, enterotoxigenic *Escherichia coli*, colon, liver

## Abstract

**Introduction:**

Infection by pathogenic bacteria during weaning is a common cause of diarrhea and intestinal inflammation in piglets. Supplementing the diet with synbiotics is beneficial for animal health. The strain of *Lactiplantibacillus plantarum* L47 (L47) isolated in our lab exhibited good probiotic properties when combined with inulin. Here, the effectiveness of combining L47 and inulin (CLN) in protecting against enterotoxigenic *Escherichia coli* (ETEC) induced colon and liver inflammation in weaned piglets was evaluated.

**Methods:**

Twenty-eight piglets aged 21 days were randomly assigned into 4 groups: CON (control), LI47 (oral CLN culture fluid, 10^10^ CFU/d of L47 and 1 g/d of inulin), ECON (oral ETEC culture fluid, 10^10^ CFU/d), and ELI47 (oral CLN and ETEC culture fluid). After 24 days, the colon and liver samples were collected for further analysis.

**Results and discussion:**

CLN alleviated colon damage caused by ETEC challenge, as evidenced by an increase of colonic crypt depth, mRNA expression of tight junction *Claudin-1* and *Occludin*, GPX activity, the concentration of IL-10 and sIgA (*p* < 0.05). Moreover, there was a decrease in MDA activity, the load of *E. coli*, the concentration of LPS, gene expression of *TLR4*, and the concentration of TNF-*α* and IL-6 (*p* < 0.05) in colonic mucosa. Additionally, CLN counteracted liver damage caused by ETEC challenge by modulating pathways associated with immunity and disease occurrence (*p* < 0.05).

**Conclusion:**

Supplementing with CLN alleviated colon inflammation induced by ETEC challenge by decreasing the *E. coli*/LPS/*TLR4* pathway and regulating hepatic immune response and disease-related pathways, suggesting that CLN could protect intestinal and liver health in animals.

## 1 Introduction

Post-weaning diarrhea in piglets presents a significant challenge for intensive pig farms, resulting in substantial economic losses due to high morbidity rates (1, 2). Reports indicate that mortality among piglets affected by post-weaning diarrhea ranges from 20 to 30% (3). Enterotoxigenic *Escherichia coli* (ETEC) is a pathogen causing diarrhea in piglets, exerting its pathogenic effects by releasing adhesins and enterotoxins (4), thereby instigating intestinal inflammation, compromising intestinal barrier function (5), and consequently reducing gut microbiota diversity (6).

Probiotics are widely used to alleviate post-weaning stress in piglets due to their environmental friendliness, safety, and effectiveness (7, 8). For example, *Lactobacillus plantarum* has been found to preserve intestinal mucosal barrier function, thereby shielding intestinal epithelia from exogenous stimuli (9). *Lactobacillus plantarum* LLY-606 restored gut microbiota dysbiosis and reduced inflammation by inhibiting the activation of the TLR4/MYD88/NF-kB signaling pathway (10). Prebiotics are utilized by specific gut microbiota, for example, *Bifidobacterium* and *Lactobacillus* metabolize them into acetate and lactate, thereby providing nourishment for other microbes (11, 12). Inulin, a soluble dietary fiber categorized as a prebiotic, is found in the roots and stems of plants such as Asteraceae and Campanulaceae, and is metabolized into short-chain fatty acids (SCFAs) by colon microbes (13). Reports indicate that inulin relies on gut microbiota to alleviate inflammation, enhance intestinal integrity, and boost host immunity (14–16). Liu et al. found that the combination of *Lactobacillus rhamnosus* and inulin increased the abundance and diversity of the colonic microbiota, enhancing the levels of beneficial bacteria such as *Lactobacillus* and *Alistipes*, and alleviating dextran sulfate (DSS) induced ulcerative colitis (17). Ayala-Monter demonstrated that the combined use of inulin and *Lactobacillus casei* increased the weaning weight of nursing lambs while reducing the abundance of *E. coli* in fecal samples and the incidence of diarrhea (18). In our previous study with mice, we observed similar results, where the combination of *Lactiplantibacillus plantarum* L47 and inulin (CLN) increased the production of SCFAs in the colon, decreased the abundance of *E. coli*, and reduced the expression of inflammatory factors, thereby alleviating DSS-induced colitis (19).

Growing evidence indicates a correlation between gut microbiota dysbiosis and the onset of metabolic liver diseases (20, 21). Lipopolysaccharides (LPS) produced by Gram-negative bacteria can reach the liver and contribute to the development of chronic hepatitis (22). Therefore, we hypothesized that CLN might mitigate damage to the colon and liver by regulating gut microbes in ETEC-challenged weaned piglets, and we aimed to preliminarily explore its mechanisms of action.

## 2 Materials and methods

This research followed the animal welfare regulations in China for animal experimentation and received authorization from the Animal Ethics Committee at Nanjing Agricultural University (SYXK-2021-0086).

### 2.1 Preparation of CLN mixture and ETEC culture fluid

The strain of L47, isolated from healthy pig intestines, was preserved in our lab (23). Its probiotic properties and synbiotic effect with inulin *in vitro* were evaluated in our previous studies (24). After thawing, the strain was inoculated into MRS liquid medium and activated at 37°C for 24 h. This activation process was repeated twice. The concentration of L47 in the activated culture was 1.0 × 10^9^ CFU/mL, then mixed with inulin at a 10:1 (v/w) ratio. Additionally, the *E. coli* K88 strain CVCC224 was provided by the China Institute of Veterinary Drugs Control, and the concentration of ETEC culture fluid was 1.0 × 10^9^ CFU/mL.

### 2.2 Animal experimental design

**Figure 1:**
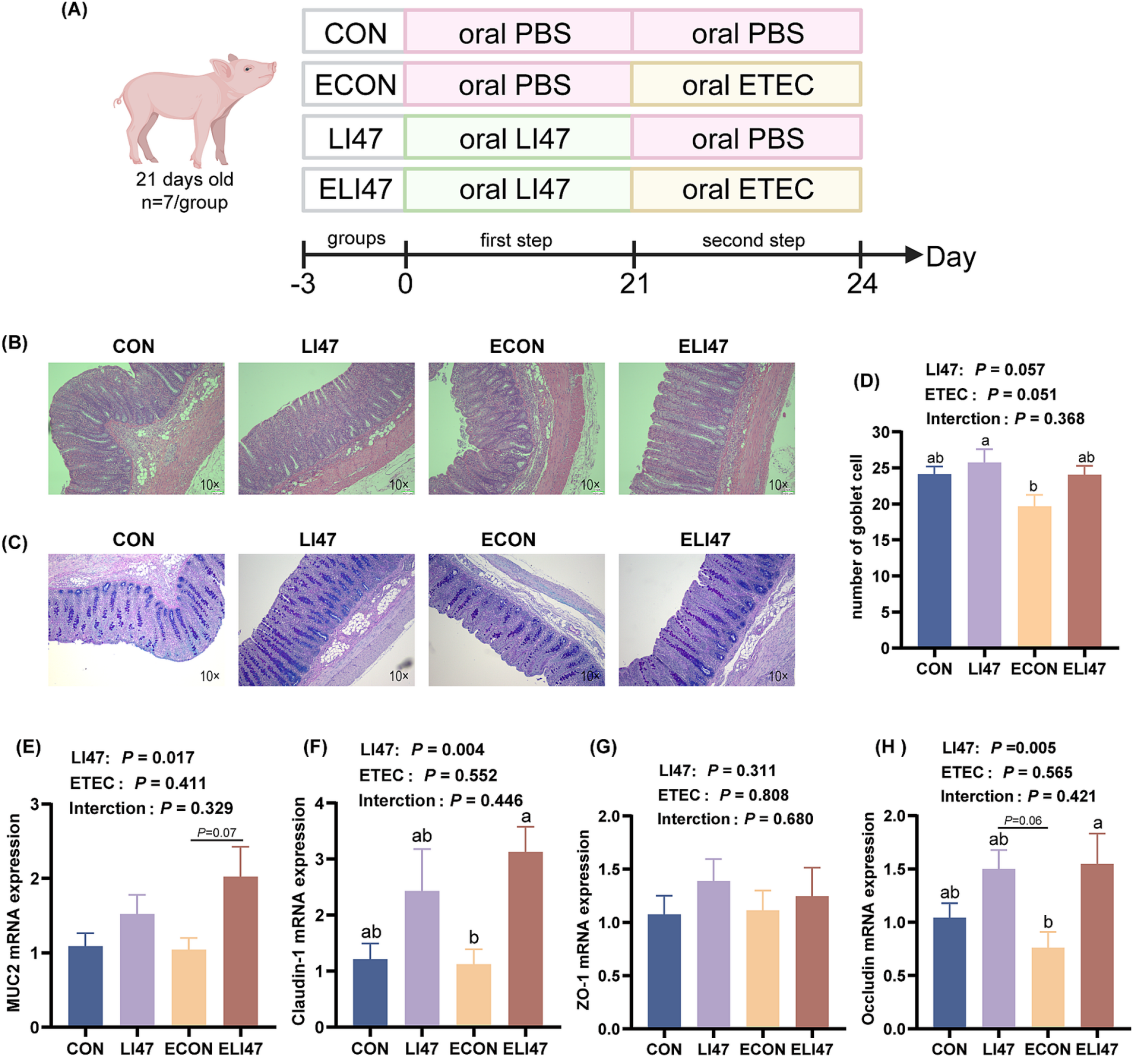
CLN alleviates colon morphology and barrier function damage. (A) Experimental design diagram. Colon tissues stained with (B) H&E and (C) AB-PAS. Scale bar, 100 μm. (D) Number of goblet cells in the colon under each field of view (*n* = 7). Gene expressions of (E) *MUC2*, (F) *claudin-1*, (G) *ZO-1* and (H) *occluding* in the colon mucosa. Results are expressed as mean ± SEM (*n* = 7), and shoulder markings without the same letter indicate differences (*p* < 0.05). CON, PBS intervention. LI47, a mixture of CLN intervention. ECON, PBS intervention and challenged by ETEC. ELI47, a mixture of CLN intervention and challenged by ETEC. The *p*-value of LI47 < 0.05 indicates a significant difference between the groups with CLN treatment (LI47 and ELI47) and those without CLN treatment (CON and ECON). The *p*-value of ETEC <0.05 indicates a significant difference between the groups treated with ETEC (ECON and ELI47) and those without ETEC (CON and LI74). The *p*-value of interaction <0.05 indicates a significant interaction between these two factors (CLN and ETEC) (as shown in the following figure).

A total of 28 castrated male piglets, aged 21 days and of the Duroc × Landrace × Yorkshire breed (6.80 ± 0.84 kg), were allocated into 4 distinct groups, designated as the CON, LI47, ECON and ELI47 groups. The experimental period was separated into two stages, totaling 24 days, and all piglets were fed a basal diet throughout the experiment. In the first stage (d 0–21), the piglets were orally administrated with 10 mL PBS (CON and ECON group) or a mixture of CLN (LI47 and ELI47 group) daily. In the second stage (d 22–24), the piglets received a single oral administration of 10 mL PBS (CON and LI47 group) or ETEC culture fluid (ECON and ELI47 group) on d 22 of the experiment. The detailed procedure is shown in Figure 1A.

### 2.3 Experimental diet and management

All piglets were free to access an antibiotic-free basic diet, which was formulated following the NRC (2012) guidelines to fulfill the nutritional needs of weaning piglets, as detailed in Supplementary Table 1 (25). The nutritional level of dry matter in the diet was measured according to the method described by Zhang (26). The piglets were raised in individual cages with controlled temperatures (27 ± 2°C) and had free access to water.

### 2.4 Sample collection

The piglets were weighed and slaughtered on d 24 of the experiment. Blood from the neck artery was collected into centrifuge tubes, centrifuged at 4°C (3,500 × g, 10 min) and the serum was carefully transferred to new cryo-tubes.

The entire colon and liver were collected, and their weight and length were measured. A 1.5 cm segment of the middle colon and 1 cm × 1 cm liver tissue sample were carefully dissected using a surgical knife and forceps, then fixed in a 4% paraformaldehyde solution for a minimum of 24 h. The liver tissue and the colonic contents were then collected. The colon tissue was gently rinsed with pre-cooled physiological saline, and the colonic mucosa was collected by scraping it using a glass slide (27, 28).

### 2.5 Serum biochemistry

The serum samples were prepared and preprocessed according to the protocol of the kit manufacturer (Angle Gene, Nanjing, China) to determine the levels of the target serum markers (Table 1).

**Table 1:** The effects on serum biochemical markers of ETEC challenged piglets with the treatment of CLN.

Items	Treatments				SEM	*p*-value		
	CON^1^	LI47^2^	ECON^3^	ELI47^4^		LI47	ETEC	Interaction
AST, U/L	42.17	34.00	46.00	31.67	5.61	0.01	0.85	0.45
ALT, U/L	26.17	20.00	25.67	21.00	2.64	<0.01	0.90	0.69
TP, g/L	59.10	61.46	58.11	58.37	2.38	0.45	0.24	0.54
ALB, g/L	32.50	35.94	32.77	35.03	2.10	0.07	0.83	0.69
GLOB, g/L	26.63	25.51	25.34	23.34	2.30	0.35	0.30	0.79
A/G	1.28	1.43	1.36	1.57	0.18	0.18	0.42	0.79
ALP, U/L	191.00	152.29	186.33	194.00	23.62	0.35	0.27	0.17
LDH, U/L	652.83^ab^	606.00^ab^	881.00^a^	523.67^b^	120.63	0.03	0.40	0.08
SUN, mmol/L	4.82	6.10	4.54	5.09	0.70	0.08	0.21	0.47
GLU, mmol/L	4.31	4.50	4.39	4.43	0.56	0.79	0.98	0.84
TC, mmol/L	2.05	1.73	1.64	1.80	0.21	0.60	0.21	0.13
TG, mmol/L	0.58	0.56	0.59	0.49	0.10	0.45	0.69	0.61

ETEC, enterotoxigenic *Escherichia coli*; AST, Aspartate aminotransferase; ALT, Alanine aminotransferase; TP, Total protein; ALB, Albumin; GLOB, Globulin; A/G, Albumin/ Globulin; ALP, Alkaline phosphatase; LDH, Lactate dehydrogenase; SUN, Serum urea nitrogen; GLU, Glucose; TC, Total cholesterol; TG, Triglycerides. Without a shared letter within a row indicate significant differences (*p* < 0.05).

1 CON, PBS intervention.

2 LI47, a mixture of CLN intervention.

3 ECON, PBS intervention and challenged by ETEC.

4 ELI47, a mixture of CLN intervention and challenged by ETEC.

### 2.6 Morphological observation

The fixed colon and liver tissue samples (as described by 2.4) were embedded in paraffin. Cross-sections of each sample were prepared and stained using either H&E or AB-PAS staining methods, then sealed with neutral resin (29, 30). Ultimately, the Image-Pro Plus 6.0 image system was utilized to measure the crypt depth and count goblet cells in the colon samples, as well as to examine the liver samples. Eight different fields were randomly selected per slide, and the average values were calculated as single-slide data.

### 2.7 Colon antioxidant activity

A 1:9 ratio of the colonic mucosa sample and pre-cooled 0.9% physiological saline were mixed and then centrifuged at 4°C (3,500 × g/10 min). The protein concentration was determined by utilizing a kit (Beyotime, Shanghai, China). Finally, the concentrations of total antioxidant capacity (T-AOC), malondialdehyde (MDA), glutathione peroxidase (GPX), catalase (CAT), and total superoxide dismutase (T-SOD) were determined following the kit instructions (Nanjing Jiancheng, China).

### 2.8 16S rRNA sequencing

The total DNA was obtained from colon digest using a kit from Omega Bio-Tek (Norcross, GA, USA), and primers 515F (5′- brocade-GTGCCGCCAGCMGCCGG-3′) and 907R (5′- CCGTCAATTCMTTTRAGT-TT-3′) were used to amplified the V4-V5 region of the 16S rRNA gene, where brocade is the unique eight base sequence for each sample. Subsequently, following the Illumina genomic DNA library preparation protocol, the merged DNA products were used to construct an Illumina paired-end library. The amplicon library was then sequenced on the Illumina MiSeq platform (Shanghai BIOZERON Co., Ltd) according to the standard protocol using paired-end sequencing (2 × 250). The Illumina PE250 sequencing reads were processed to obtain valid sequences for all samples based on the barcode, and quality control filtering was performed on the reads, followed by optimization and statistical analysis of the data. Operational taxonomic units (OTUs) were defined at a 97% similarity level for bioinformatics statistical analysis and alpha diversity assessment.

### 2.9 Colon short-chain fatty acids

A previously described method was used to determine the contents of SCFAs (31). Firstly, the pre-treated sample was mixed with a 25% (w/v) metaphosphate acid solution and then stored overnight at −20°C. After thawing, the mixture was centrifuged to obtain the supernatant, which was then filtered through a 0.22 μm filter.

### 2.10 RNA-seq analysis in liver

According to the manufacturer’s instructions (Invitrogen), total RNA was extracted from liver tissue using TRIzol reagent, followed by the removal of rRNA and enrichment of mRNA. The Illumina TruSeq™ RNA Sample Prep Kit was then employed to construct a cDNA library by reverse transcribing the mRNA into cDNA. The purified double-stranded cDNA underwent end repair, A-tailing, and adapter ligation, followed by the selection of approximately 200 bp cDNA fragments. These fragments were PCR amplified and purified again to obtain the final library. Quality control of the library was performed using agarose gel electrophoresis, and RNA concentration, purity, and integrity were assessed. After passing quality control, sequencing was conducted using the Illumina TruSeq SBS Kit (300 cycles). The raw data underwent quality control to generate valid data, which were aligned to the reference genome to identify differentially expressed genes (DEGs, *p* < 0.05) among the samples. The Kyoto Encyclopedia of Genes and Genomes (KEGG) was used for annotation and functional enrichment of the DEGs.

### 2.11 Enzyme-Linked Immunosorbent Assay

The concentrations of immune cytokines, tumor necrosis factor (TNF)-*α*, secretory immunoglobulin A (sIgA), and LPS in the colonic mucosa were determined using ELISA kits supplied by Nanjing Jiancheng (Nanjing, China).

### 2.12 Quantitative real-time PCR

Total RNA was extracted from both colonic mucosa and liver tissue using a kit from Accurate (Hunan, China), and the RNA quality was assessed using the ND-2000 (Thermo Scientific, Wilmington, United States). Subsequently, PCR detection was performed using a kit (Accurate, Hunan, China). The primers utilized in the study are listed in Table 2. Finally, the expression levels of the target genes were assessed using the 2^-△△Ct^ method (32).

**Table 2:** Primer sequences of RT-PCR.

Gene	Forward primer (5′–3′)	Reverse primer (5′–3′)
*MUC2*	AGGACGACACCATCTACCTCA	TGTTCCACACGAGAGCAAGG
*ZO-1*	TCTGCCGAGACAACAGCATC	CAGGAGTCATGGACGCACAG
*Occludin*	AGCAGTGGTAACTTGGAGGC	CAGTCTTCCTCCAGCTCGTC
*Claudin-1*	CTATGACCCCATGACCCCAG	GGCCTTGGTGTTGGGTAAGA
*TNF-α*	CCTTCCACCAACGTTTTCCTC	AGTCGATCATCCTTCTCCAGC
*IL-1β*	CTCTCCAGCCAGTCTTCATTG	ATTATTGTTGTCACCGTAGTTAGC
*IL-6*	GGCCATTCGGATAATGTAGCT	GTGTCCTAACGCTCATACTTT
*IL-10*	CCTGACTGCCTCCCACTTTC	GGGCTCCCTAGTTTCTCTTCCT
*TLR4*	TGACAACATCCCCACATCAGT	TTCCCGTCAGTATCAAGGTGG
*E. coli*	CATGCCGCGTGTATGAAGAA	CGGGTAACGTCAATGAGCAAA
β-Actin	GGACCTGACCGACTACCTCA	CCATCTCCTGCTCGAAGTCC

MUC2, Mucin 2; ZO-1, Zonula occludens 1; TNF-α, Tumor necrosis factor; IL-1β, Interleukin 1 beta; IL-6, Interleukin 6; IL-10, Interleukin 10; TLR4, Toll like receptor 4.

### 2.13 Statistical analysis

Presented the data as mean ± standard error of the mean (SEM) and analyzed by SPSS 25.0 software (SPSS Inc., Chicago, IL, United States). *p*-value <0.05 means statistical difference, 0.05 < *p* < 0.10 means a trend. The general linear model (GLM) procedure with a two-factor (LI47 and ETEC) analysis of variance (ANOVA) design was employed. Significant differences were conducted by Tukey’s multiple range test. Different bacterial populations were compared using the Mann–Whitney U test, and multiple comparisons were corrected using the Benjamini-Hochberg false discovery rate. Finally, graphs were formed using GraphPad Prism 8.0.2 (La Jolla, CA, United States).

## 3 Results

### 3.1 CLN improves colon morphology induced by ETEC-challenged

As shown in Table 3, treatment with CLN did not affect either colonic length or weight. Figures 1B,C shows that ETEC challenge decreased the colonic crypt depth, while treatment with CLN increased it (*p* < 0.05). An interaction effect on crypt depth was observed between ETEC challenge and treatment with CLN (*p* < 0.05).

**Table 3:** The effects on the intestinal morphology of ETEC-challenged piglets with the treatment of CLN.

Items	Treatments				SEM	*p*-value		
	CON^1^	LI47^2^	ECON^3^	ELI47^4^		LI47	ETEC	Interaction
Colon length, cm	166.21	216.00	178.64	176.98	27.12	0.22	0.50	0.19
Colon weight, g	46.68	50.82	51.56	46.65	6.90	0.78	0.90	0.48
Crypt depth, μm	589.91^a^	513.73^ab^	461.20^b^	553.86^a^	38.20	0.63	<0.01	<0.01

ETEC, enterotoxigenic *Escherichia coli*. Without a shared letter within a row indicate significant differences (*p* < 0.05).

1 CON, PBS intervention.

2 LI47, a mixture of CLN intervention.

3 ECON, PBS intervention and challenged by ETEC.

4 ELI47, a mixture of CLN intervention and challenged by ETEC.

### 3.2 CLN enhances colon barrier function

The goblet cell count was decreased by ETEC challenge, but treatment with CLN increased it (Figure 1D, *p* > 0.05). Additionally, treatment with CLN increased the mRNA expression of *MUC2* (Figure 1E, 0.05 < *p* < 0.1), *Occludin* and *Claudin-1* (Figures 1F–H, *p* < 0.05). No interaction was found in the goblet cell count and the mRNA expression of *MUC2*, *Occludin*, *Claudin-1*, and *ZO-1* between ETEC challenge and CLN treatment (*p* > 0.05).

### 3.3 CLN alleviates colonic inflammation in ETEC-challenged piglets

**Figure 2:**
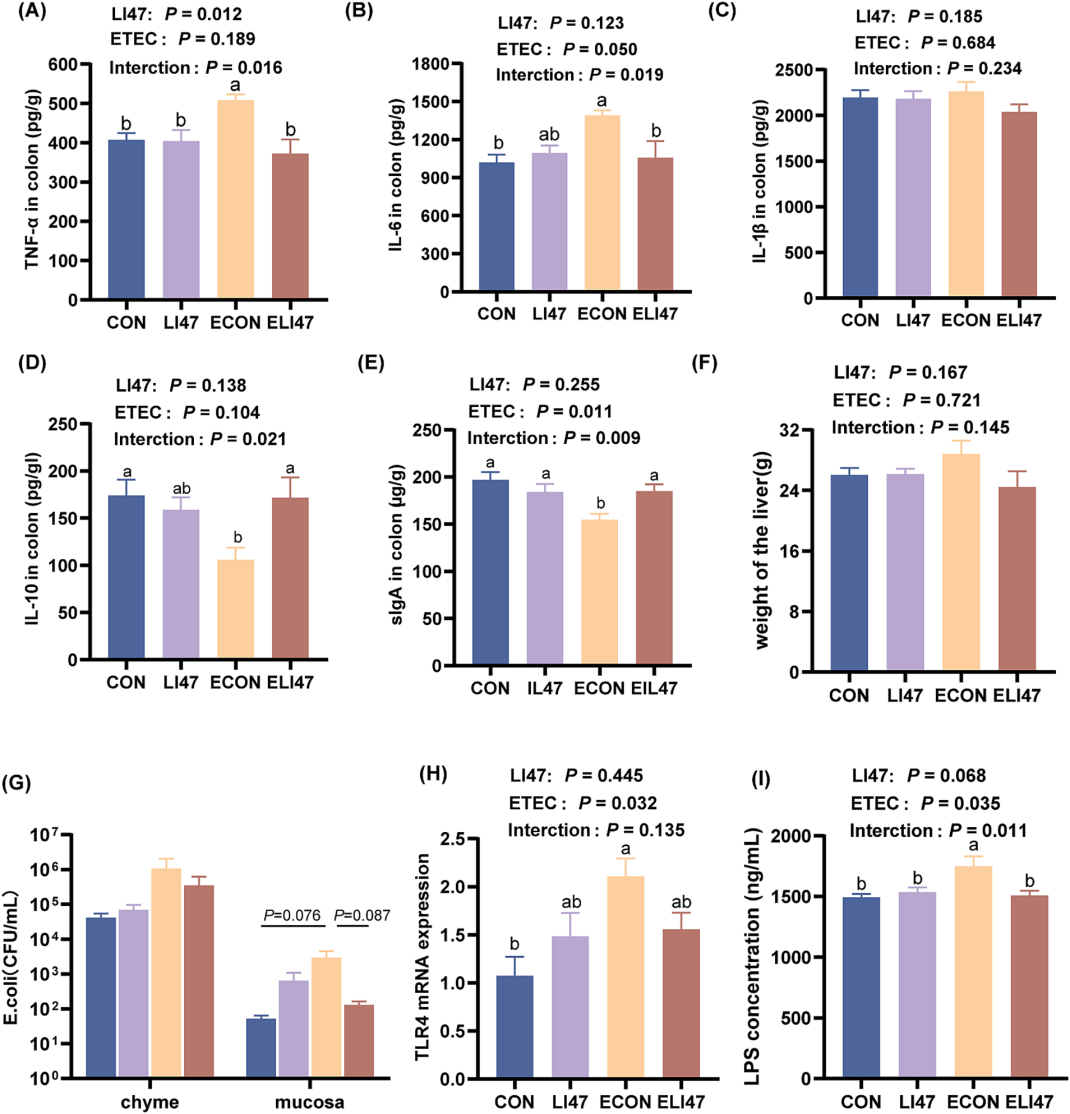
Effects of the treatment with CLN on the cytokines and the level of *E. coli*/LPS/*TLR4* in the colon mucosa and the liver weight. Concentration of (A) TNF-*α*, (B) IL-6, (C) IL-1*β*, (D) IL-10, (E) sIgA in the colon mucosa. (F) The weight of the liver. (G) The colonization level of *E. coli* in colon chyme and mucosa. (H) The concentration of LPS and (I) gene expression of *TLR4* in colon mucosa. Results are expressed as mean ± SEM (*n* = 7), and shoulder markings without the same letter indicate differences (*p* < 0.05). CON, PBS intervention. LI47, a mixture of CLN intervention. ECON, PBS intervention and challenged by ETEC. ELI47, a mixture of CLN intervention and challenged by ETEC.

ETEC challenge increased the concentration of TNF-*α* and IL-6 in the colonic mucosa (*p* < 0.05), while treatment with CLN showed the opposite effect (Figures 2A,B, *p* < 0.05). Additionally, ETEC challenge decreased the concentration of IL-10 and secretory immunoglobulin A (sIgA) in the colonic mucosa, whereas treatment with CLN increased their levels (Figures 2D,E, *p* < 0.05). No difference was observed in IL-1β across the four groups (Figure 2C, *p* > 0.05). An interaction effect on the concentration of TNF-α, IL-6, IL-10, and sIgA was found between treatment with CLN and ETEC challenge (*p* < 0.05), while no interaction was observed in IL-1β (*p* > 0.05).

### 3.4 CLN improves the colonic antioxidant capacity in ETEC-challenged piglets

As shown in Table 4, ETEC challenge decreased the concentration of GPX (*p* < 0.05) and increased MDA (*p* < 0.05), both of which were reversed by treatment with CLN (*p* < 0.05). Additionally, ETEC challenge decreased the concentration of CAT. There was an interaction found in the concentration of MDA between treatment with CLN and ETEC challenge (*p* < 0.05), while no interaction was found on T-AOC, T-SOD, GPX, and CAT (*p* > 0.05).

**Table 4:** CLN enhances the antioxidant properties of colon mucosa in ETEC-challenged piglets.

Items	Treatments				SEM	*p*-value		
	CON^1^	LI47^2^	ECON^3^	ELI47^4^		LI47	ETEC	Interaction
T-AOC, mM	0.38	0.45	0.43	0.42	0.04	0.14	0.56	0.18
GPX, U·mg^−1^ prot	9.90^b^	14.06^a^	5.51^c^	9.34^b^	1.25	<0.01	<0.01	0.85
T-SOD, U·mg^−1^ prot	557.70	565.25	589.81	589.70	15.34	0.74	0.02	0.73
CAT, U·mg^−1^ prot	4.36^a^	4.11^a^	2.75^b^	2.71^b^	0.47	0.67	<0.01	0.75
MDA, mol·mg^−1^ prot	1.00^b^	1.41^b^	2.17^a^	1.56^b^	0.20	0.50	<0.01	<0.01

ETEC, enterotoxigenic *Escherichia coli*; T-AOC, total antioxidant capacity; GPX, glutathione peroxidase; T-SOD, total superoxide dismutase; CAT, catalase; MDA, malondialdehyde. Without a shared letter within a row indicate significant differences (*p* < 0.05).

1 CON, PBS intervention.

2 LI47, a mixture of CLN intervention.

3 ECON, PBS intervention and challenged by ETEC.

4 ELI47, a mixture of CLN intervention and challenged by ETEC.

### 3.5 CLN improves the colonic microbiota in ETEC-challenged piglets

**Figure 3:**
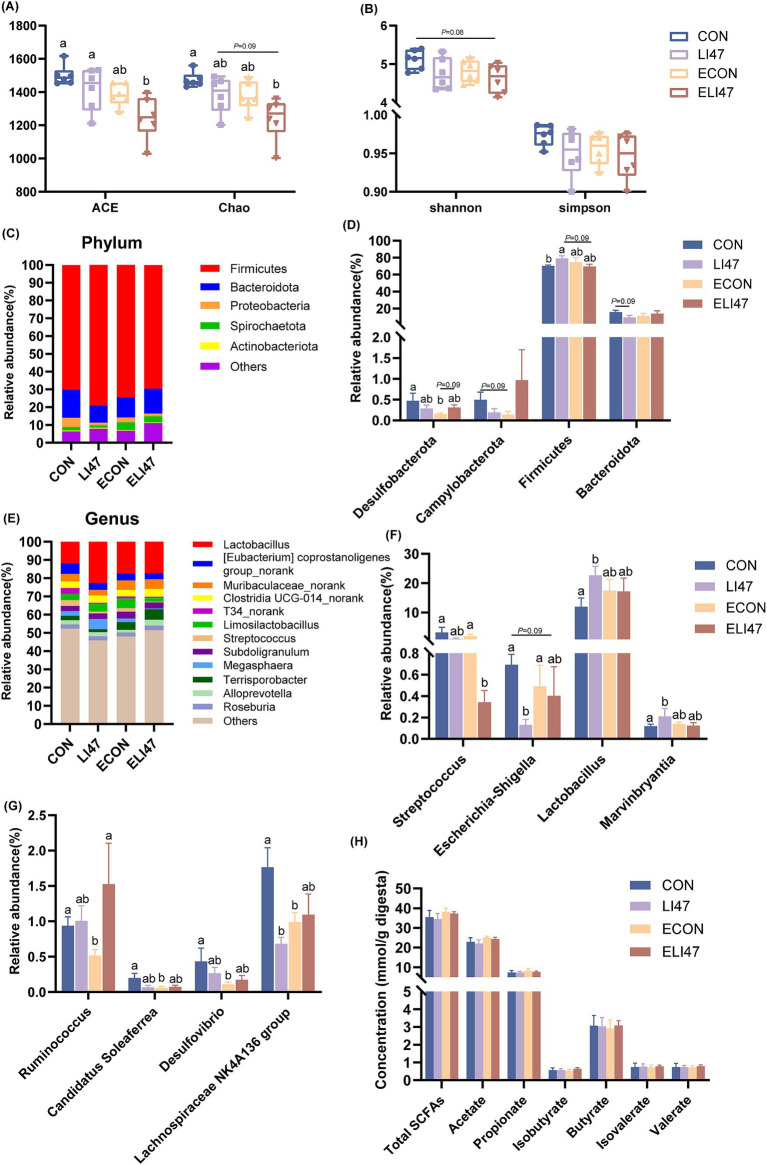
CLN regulates the composition of colonic digest microbiota and SCFAs concentration in piglets. (A) ACE, Chao, (B) Shannon and Simpson index among four groups. Relative abundance of the microbial (C) phylum and (E) genus, (D) different phyla and (F–G) different genera. (H) The concentrations of SCFAs among four groups. Results are expressed as mean ± SEM (*n* = 7), and shoulder markings without the same letter indicate differences (*p* < 0.05). CON, PBS intervention. LI47, a mixture of CLN intervention. ECON, PBS intervention and challenged by ETEC. ELI47, a mixture of CLN intervention and challenged by ETEC.

In the ELI47 group, ACE and Chao index were lower compared to the CON and LI47 groups (Figure 3A, *p* < 0.05). However, no differences were observed in Shannon and Simpson index across the 4 groups (Figure 3B, *p* > 0.05). Firmicutes and Bacteroidota were the most abundant phylum (Figure 3C). Treatment with CLN increased the relative abundance of Firmicutes (*p* < 0.05) while showing a trend toward decreasing the level of Bacteroidota (Figure 3D, 0.05 < *p* < 0.10). The proportions of Desulfobacterota and Campylobacterota were decreased by ETEC challenge (Figure 3D, 0.05 < *p* < 0.10), while CLN treatment showed an increasing trend of Desulfobacterota (Figure 3D, 0.05 < *p* < 0.10).

*Lactobacillus* was the most abundant genus in the colon (Figure 3E), and CLN treatment promoted the proportions of *Lactobacillu*s and *Marvinbryantia* (Figure 3F, *P* < 0.05), while decreasing the levels of *Lachnospiraceae NK4A136 group*, *Escherichia-Shigella* and *Streptococcus* (Figures 3F,G, *p* < 0.05). Lower abundances of *Ruminococcus*, *Candidatus Soleaferrea*, *Desulfovibrio*, and *Lachnospiraceae NK4A136 group* were observed following ETEC challenge (Figure 3G, *p* < 0.05). However, no differences in the SCFAs were found among the 4 groups (Figure 3H, *p* > 0.05).

### 3.6 CLN inhibits the *Escherichia coli*/LPS/TLR4 pathway in ETEC-challenged piglets

ETEC challenge resulted in an increased *E. coli* load (0.05 < *p* < 0.1), a higher LPS concentration (*p* < 0.05), and an elevated level of *TLR4* (*p* < 0.05) in the colonic mucosa (Figures 2G–I), while these were decreased in ELI47 group (0.05 < *p* < 0.10). However, there was no difference in the load of *E. coli* in the colonic digest among the 4 groups (Figure 2G, *p* > 0.05).

### 3.7 CLN alleviates the expression of liver injury markers in serum in ETEC-challenged piglets

Compared with the ECON group, the concentration of LDH (*p* < 0.05) and AST (*p* = 0.08) decreased in the ELI47 group (Table 1). However, there was no difference in the concentrations of ALT, TP, TC, TG, SUN, etc. among the four groups (Table 1, *p* > 0.05). An interaction was revealed in LDH level between ETEC-challenge and treatment with CLN (*p* < 0.05).

### 3.8 CLN has no effects on liver morphology and inflammation

**Figure 4:**
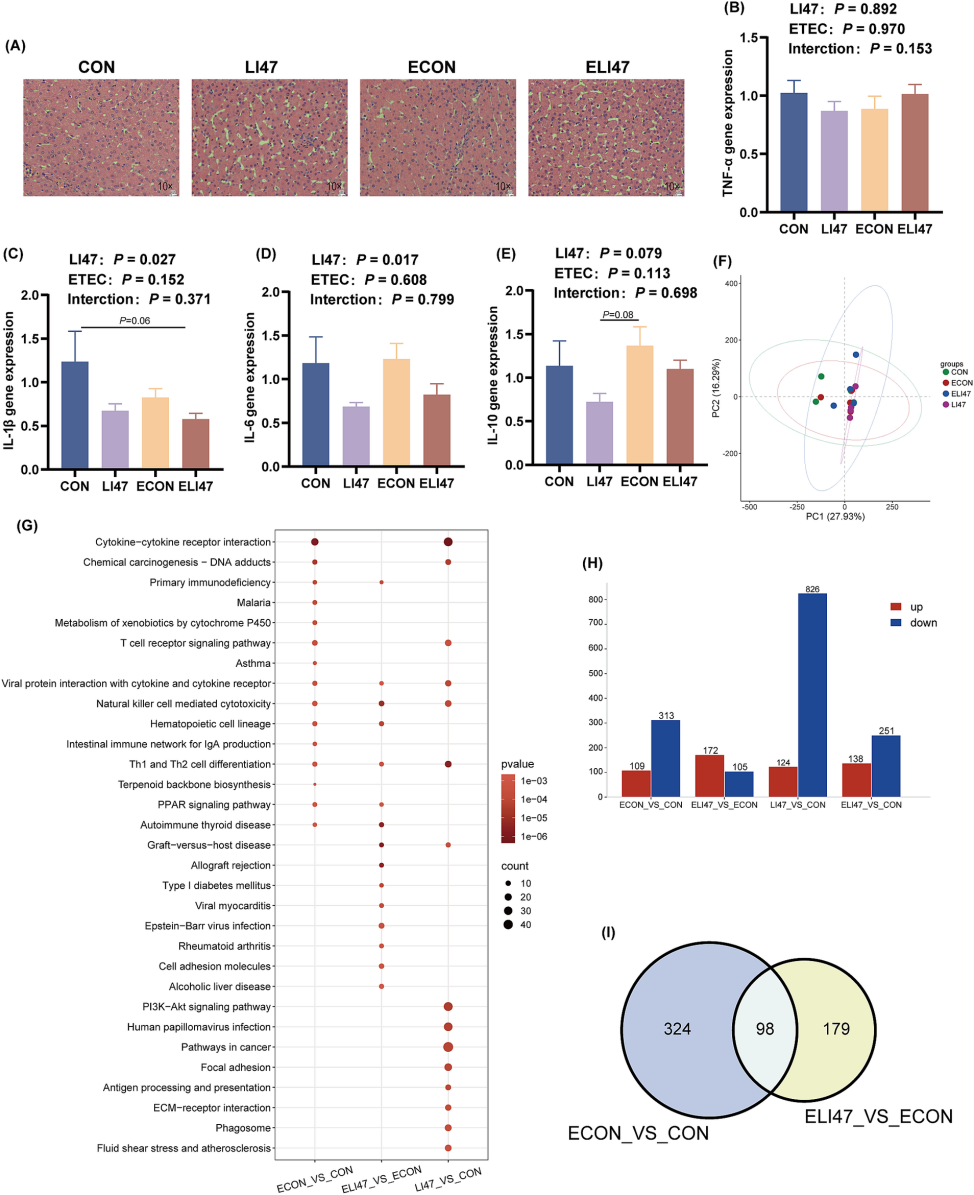
Effects of the treatment with CLN on the liver morphology and RNA-seq of ETEC-challenged piglets. (A) Liver tissues stained with H&E. Scale bar, 100 μm. Gene expression of (B) TNF-α, (C) IL-1β, (D) IL-6, (E) IL-10 in liver tissues (*n* = 7). (F) PCA analysis, (H) DEGs, (I) Venn diagram and (G) KEGG pathway enrichment analysis of DEGs among four groups (*n* = 4). Results are expressed as mean ± SEM. CON, PBS intervention. LI47, a mixture of CLN intervention. ECON, PBS intervention and challenged by ETEC. ELI47, a mixture of CLN intervention and challenged by ETEC.

There were no differences in liver weight and liver tissue sections among the 4 groups, as all exhibited intact liver tissue structure with clear and complete hepatic lobules and hepatic cords, well-arranged hepatocytes, and no visible lipid droplet accumulation (Figures 2F, 4A). Additionally, the mRNA expression of *TNF-α*, *IL-1β*, *IL-6*, and *IL-10* showed no differences among the 4 groups (Figures 4B–E*, p* > 0.05). No interaction was observed on *TNF-α*, *IL-1β*, *IL-6*, and *IL-10* levels between ETEC-challenged and the supplementation of CLN (*p* > 0.05).

**Figure 5:**
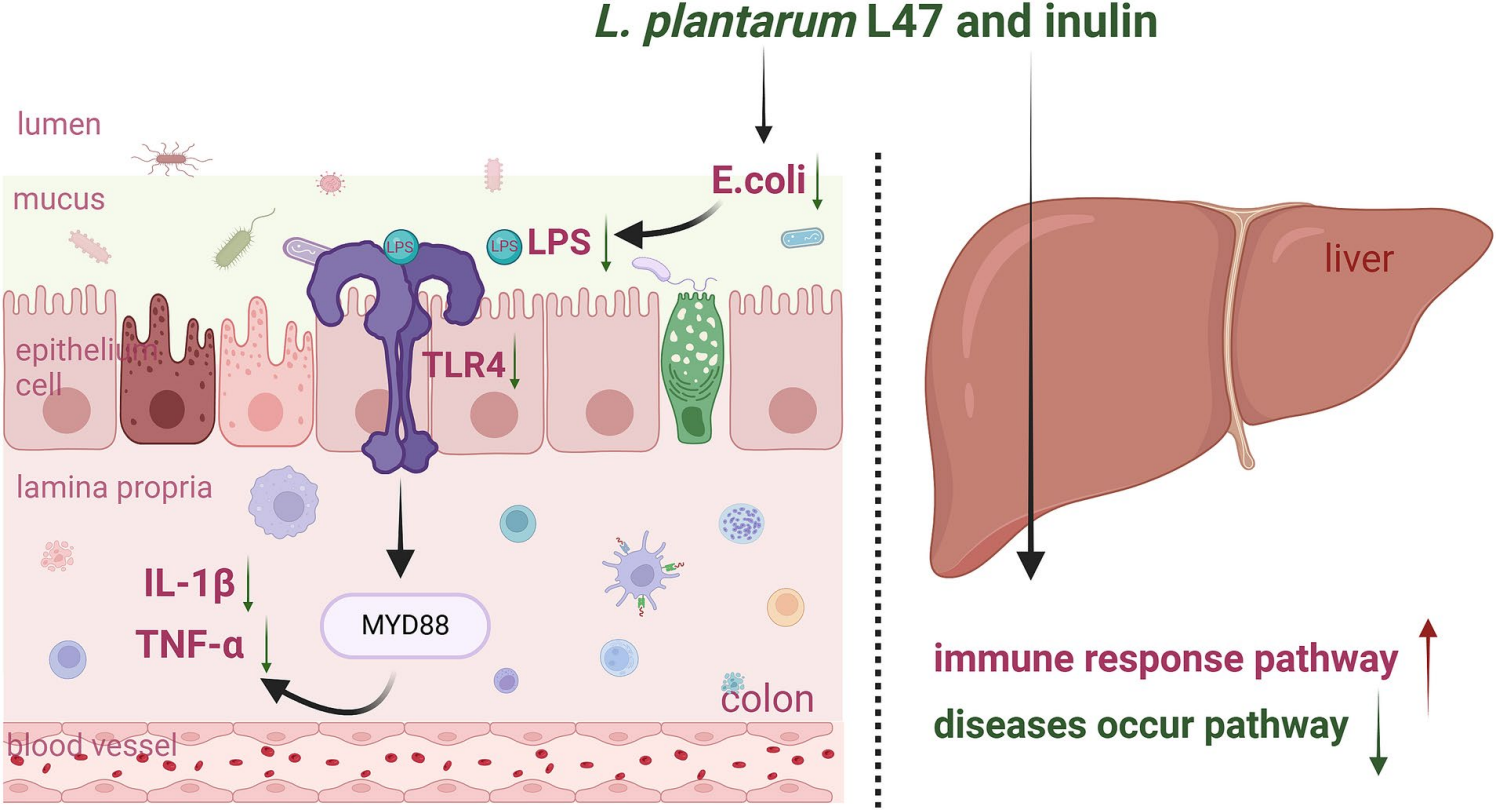
Graphic Summary: Effects of the treatment with CLN on the colon and liver inflammation of ETEC-challenged piglets (by Biorender). LI47, *L. plantarum* L47 and inulin; *E. coli, Escherichia coli*; LPS, lipopolysaccharide; TLR4, toll like receptor 4; MYD88, myeloid differentiation primary response gene (88); IL, interleukin; TNF-α, tumor necrosis factor-α.

### 3.9 CLN regulates pathways related to immunity and disease occurrence

The PCA analysis presented no obvious separation trend among the 4 groups (Figure 4F). ETEC challenge resulted in 422 differentially expressed genes (DEGs) (109 up-regulated, 313 down-regulated) compared to the CON group (Figure 4H), while treatment with CLN (ELI47 group) showed 277 DEGs (172 up-regulated, 105 down-regulated) (Figure 4H). Furthermore, 940 DEGs (124 upregulated, 826 downregulated) were identified in the LI47 group compared to the CON group (Figure 4H). Both ETEC-challenge and treatment with CLN modulated 98 DEGs (Figure 4I). KEGG enrichment analysis based on DEGs was performed (Figure 4G). Pathways associated with immune regulation, including Hematopoietic cell lineage, Intestinal immune network for IgA production, and Th1 and Th2 cell differentiation were down-regulated by ETEC challenge (Figure 4G). Besides, treatment with CLN up-regulated pathways such as Th1 and Th2 cell differentiation, Hematopoietic cell lineage, while down-regulating pathways like Alcoholic liver disease, Focal adhesion, and Fluid shear stress and atherosclerosis, etc. (Figure 4G).

## 4 Discussion

The intestinal barrier, formed by numerous epithelial cells, serves the crucial role of distinguishing the external environment from the internal host system (33). The intestine is a complex and crucial organ involved in digesting and absorbing nutrients, managing metabolic processes, and regulating immune functions (34). However, ETEC infection induces intestinal damage such as villous atrophy, inflammation, oxidative stress, and dysbiosis (5). Therefore, it is of practical significance to explore effective strategies to protect intestinal health. Synbiotics, which are composed of probiotics and prebiotics, maintain or enhance intestinal health through various mechanisms (35–37). Here, we aim to explore the potential benefits of dietary CLN supplementation against ETEC colonic injury.

The function of intestinal morphology and the integrity of the barrier are closely associated with the occurrence of intestinal inflammation and oxidative stress (38–43). The intestine is lined with numerous intestinal epithelial cells (IECs) that secrete mucins (such as MUC2) and antimicrobial proteins to maintain the chemical barrier, while intercellular tight junctions maintain the physical barrier of the intestine (33, 44, 45). Alexia’s study suggests that the synbiotic combination of *Lactobacillus acidophilus* W37 and inulin can directly stimulate IECs or immune cells, thereby activating immune receptors involved in immune regulation (46). In this study, ETEC-challenge induced colonic injury, while CLN supplementation exhibited protective and restorative effects. This is consistent with previous research (17, 47, 48), indicating that CLN has the potential to protect intestinal health, and its protective mechanism warrants further investigation.

Recently, there has been a growing interest in altering the intestinal microbiota using synbiotics (22, 49, 50). Robin supplemented piglets’ diets with *Pediococcus acidilactici* and lactulose, which increased the proportion of beneficial microbes like *Lactobacillus* and *Prevotella*, and alleviated the intestinal inflammation induced by Shiga toxin-producing *Escherichia coli* (51). Similarly, our previous study indicated that intervention with CLN altered the fermentation environment of the simulated pig colon and increased the production of SCFAs *in vitro* (24). Firmicutes are Gram-positive bacteria, including the genera *Bacillus*, *Clostridium*, and *Lactobacillus* (52). The increase in abundance of Firmicutes by supplementing CLN may be attributed to the increase in abundance of *Lactobacillus*, it reported that the *Lactobacillus* genus promoted intestinal epithelial cell proliferation, maintained intestinal barrier function, and reduced intestinal inflammatory responses (53, 54). *Lachnospiraceae* are considered a beneficial bacterium in the gut in numerous studies, involved in fiber digestion and SCFAs production, however, *Lachnospiraceae* may also be associated with the occurrence of metabolic diseases (55–57). In addition, supplementation with CLN decreased the proportion of harmful bacteria like *Escherichia-Shigella* and *Streptococcus* in the colon. This suggests that CLN might exert anti-inflammatory and antioxidant properties through its impact on intestinal microbiota composition. Several signaling pathways are involved in regulating inflammation and oxidative stress, including AHR/HTAT3 (58), TLR4/NOD (19), AHR/Nrf2 (59), and NF-kB/MAPK (60). LPS is a component released upon the death of Gram-negative bacteria like *E. coli*, and it plays a role in inflammation and can even induce sepsis (61, 62). The recognition and binding of LPS to the TLR4 receptor can activate intracellular signaling via MyD88-dependent or -independent pathways, thereby inducing inflammation (63, 64). In this study, ETEC-challenge induced increases in the expression of *E. coli*/LPS/TLR4, while CLN supplementation reduced these levels. This suggests that treatment with CLN may alleviate colonic inflammation by decreasing *E. coli* abundance in colonic mucosa and inhibiting LPS/*TLR4* recognition and binding.

LDH is present in various organs, including the liver, heart, lymph nodes, spleen, lungs, and pancreas. It participates in the glycolytic pathway and catalyzes the redox reaction between pyruvate and lactate. When these tissues are damaged, LDH is released into the bloodstream in large quantities (65, 66). Therefore, the elevated serum concentration of LDH in the ECON group may indicate that an organ has suffered damage. Interestingly, the treatment with CLN could prevent such damage from occurring. The liver is a crucial metabolic organ, and AST and ALT are commonly used as biomarkers for liver injury (67). Compared to the ECON group, the ELI47 group exhibited a trend toward reduced AST concentration, suggesting that CLN may have a protective effect against liver injury. Currently, an increasing number of studies are focusing on the relationship between gut microbiota and liver diseases (68). For instance, excessive exposure to copper has been shown to impair intestinal barrier function and disrupt microbial communities, leading to increased production of LPS and activation of the TLR4/NF-kB signaling pathway, resulting in liver inflammation (69). Additionally, Chen demonstrated that *Lactobacillus plantarum* Lp2 could inhibit LPS-induced liver injury (70). Consistent with these studies, treatment with CLN demonstrates the ability to inhibit LPS/*TLR4* production and has the potential to prevent liver injury. To further explore the effects of CLN on the liver, we conducted transcriptome analysis, which revealed significant enrichment of the Th1 and Th2 cell differentiation pathways. Th1 cells primarily induce the production of interferon-*γ* (IFN-γ), which is critical for the host’s autoimmune response. Th2 cells induce the secretion of cytokines such as IL-4, IL-5, and IL-13, promoting the production of immunoglobulin (Ig) A and Ig E, thereby regulating humoral immune responses and allergic diseases (71, 72). It is noteworthy that the balance between Th1 and Th2 cells is crucial for maintaining a normal immune response (73). A study indicated that ginsenoside Rh2 regulates Th1 differentiation and the Th1/Th2 immune balance through LDC (74). In this study, ETEC challenge downregulated the pathway of Th1 and Th2 cell differentiation, while CLN treatment upregulated this pathway. Additionally, CLN treatment inhibited several signaling pathways associated with disease development. The transcriptome results suggest that CLN may play a role in maintaining normal immune responses and inhibiting disease development. However, further studies are needed to validate the roles of these pathways. In conclusion, this study demonstrates that treatment with CLN can serve as an effective protective measure for promoting animal health.

## 5 Conclusion

Supplementation with CLN may reduce colonic inflammation induced by ETEC in weaned piglets by inhibiting the *E. coli*/LPS/*TLR4* pathway (Figure 5). Neither ETEC challenge nor the intervention of CLN impacted the liver phenotype, such as maintaining intact liver tissue structure, well-arranged hepatocytes, and no visible lipid droplet accumulation. However, both interventions presented effects on LDH and AST levels in the serum and modulated pathways associated with immunity and disease occurrence in the liver.

## Data Availability

The datasets of 16S rRNA gene sequence and transcriptome sequence presented in the study are deposited in the NCBI repository, accession number are PRJNA1185907 and PRJNA1185914.
