# Supplementary material for: Lactiplantibacillus plantarum L47 and inulin affect colon and liver inflammation in piglets challenged by enterotoxigenic Escherichia coli through regulating gut microbiota
Source: Front Vet Sci. 2024 Nov 27;11:1496893. doi: 10.3389/fvets.2024.1496893 (PMC11631943; doi:10.3389/fvets.2024.1496893)
Supplement: Supplementary file 1 [file Table_1.docx]

**Supplemental Table 1.** Basal diet composition and nutrient levels in the experiment (%, as-fed basis).

| Ingredients | Contents | Chemical composition (%) | Contents |
| --- | --- | --- | --- |
| Corn | 46.40 | Moisture^2^ | 11.83 |
| Soybean meal | 20.00 | Crude protein^2^ | 23.30 |
| Extruded soybean | 10.00 | Crude lipid^2^ | 8.06 |
| Fish meal | 10.00 | Crude fiber^2^ | 3.86 |
| Whey powder | 5.00 | Ash^2^ | 8.97 |
| Soybean oil | 4.00 | Nitrogen-free extract^3^ | 42.67 |
| L-Lysine | 0.50 | Carbohydrate^4^ | 46.53 |
| DL-Methionine | 0.05 | Gross energy^2^, kJ/g dry matter | 20.63 |
| L-Threonine | 0.05 |  |  |
| Premix^1^ | 4.00 |  |  |
| Total | 100.00 |  |  |

^1^The premix provided the following per kg of diet: vitamin A, 9600 IU; vitamin D3, 3,200 IU; vitamin E, 20mg; vitamin K3, 0.6 mg; vitamin B1, 1.6 mg; vitamin B2, 2.4 mg; vitamin B6, 1.2 mg; vitamin B12, 0.02 mg; niacin, 20 mg; pantothenic acid, 12 mg; folic acid, 0.6 mg; biotin, 0.08 mg; choline chloride, 500 mg; Fe (from ferrous sulfate), 120 mg; Cu (from copper sulfate), 60 mg; Mn (from manganese sulfate), 12 mg; Zn (from zinc sulfate), 100 mg; I (from calcium iodate), 0.4 mg; Se (from sodium selenite), 0.3 mg.

^2^These value were measured.

^3^Nitrogen-free extract = 100 – (moisture + crude protein + crude lipid +crude fber + ash).

^4^Carbohydrate = nitrogen-free extract + crude fiber.
